# The mitochondrial genome of the ambush bug *Carcinochelis bannaensis* (Hemiptera: Reduviidae)

**DOI:** 10.1080/23802359.2018.1507652

**Published:** 2018-10-29

**Authors:** Tianye Linghu, Yisheng Zhao, Fan Song, Wanzhi Cai

**Affiliations:** Department of Entomology and MOA Key Lab of Pest Monitoring and Green Management College of Pant Protection, China Agricultural University, Beijing, China

**Keywords:** Mitochondrial genome, reduviidae, phymatinae, *Carcinochelis bannaensis*

## Abstract

The complete mitochondrial genome (mitogenome) of the ambush bug, *Carcinochelis bannaensis*, was determined in this study. The sequenced mitogenome is a typical circular DNA molecule of 15,335 bp, containing 13 protein-coding genes, two rRNA genes, 22 tRNA genes and a putative control region. All protein-coding genes initiate with ATN codons and terminate with TAA codons except for *COII,COIII* and *ND5* use a single T residue as the stop codon. All tRNAs have the clover-leaf structure except for the *tRNA^Ser(AGN)^* and the length of them range from 61 to 71 bp. The control region is 797 bp long with an A + T content of 66.3%. The phylogenetic analysis result supports the monophyly of Phymatinae.

The ambush bug Phymatinae (Hemiptera: Reduviidae) is a diverse clade of predators and distinguished by the cryptic hunting behavior and morphologically diverse raptorial forelegs (Masonick et al. 2017). The genus *Carcinochelis* includes seven species distributed in the Oriental region of China. We sequenced the complete mitogenome of *Carcinochelis bannaensis*, the only one species which reaches the south of China in this genus (Cui et al. 2006). Voucher specimen (No. VCim-00115) was deposited at the Entomological Museum of China Agriculture University and the sequence was deposited in GenBank under the accession number KY069957.

The mitochondrial genome is a typical circular DNA molecule of 15,335 bp in size that encode 37 genes (13 protein-coding genes, 22 tRNA genes, and two rRNA genes) and a control region. Gene order is identical to the putative ancestral gene arrangement (Cameron 2014; Song et al. 2016; Li et al. 2017). Except for the control region, this mitogenome has seven inter-genic regions, which range from 1 to 55 bp in size. There are totally 60 bp overlapped nucleotides between neighboring genes in 15 locations, ranging from 1 to 17 bp in size.

The nucleotide composition of the whole mitogenome shows significantly AT bias. The A + T content is 70.7% with positive AT-skew (0.14) and negative GC-skew (–0.16). All protein-coding genes initiate with ATN as the start codon (four with ATT, five with ATG and four with ATA). The stop codon TAA is assigned to 10 protein-coding genes. Whereas *COII,COIII* and *ND5* use a single T residue as incomplete stop codon which is commonly reported in true bug mitogenomes (Li et al. 2012). Among tRNA genes, only *tRNA^Ser(AGN)^* cannot exhibit the classic cloverleaf secondary structure, due to the deficiency of the dihydrouridine (DHU) arm which is typical feature of insect mitogenomes (Jiang et al. 2016).

The length of the 22 sequenced tRNA genes range from 61 to 71 bp with the classic cloverleaf secondary structure. The *lrRNA* is 1242 bp long with an A + T content of 73.2%, and the *srRNA* is 761 bp long with an A + T content of 72.7%. The control region, which is located between *srRNA* and *tRNA^Ile^*, is 797 bp long and is biased toward A + T (66.3%). Except the control region, another 55 bp noncoding region located between *tRNA^Ile^* and *tRNA^Gln^*, which has also been found in other ambush bugs.

Phylogenetic tree based on the maximum likelihood method shows Phymatinae is monophyletic, which is also recovered in previous molecular and morphological analyses (Weirauch and Munro 2009; Weirauch et al. 2014) (Figure 1). The genus *Carcinochelis* is more closed to the genus *Carcinocoris*. The present data are largely congruent with recently published hypotheses in supporting paraphyly of Reduviinae (Hwang and Weirauch 2012; Liu et al. 2018).

**Figure 1.**
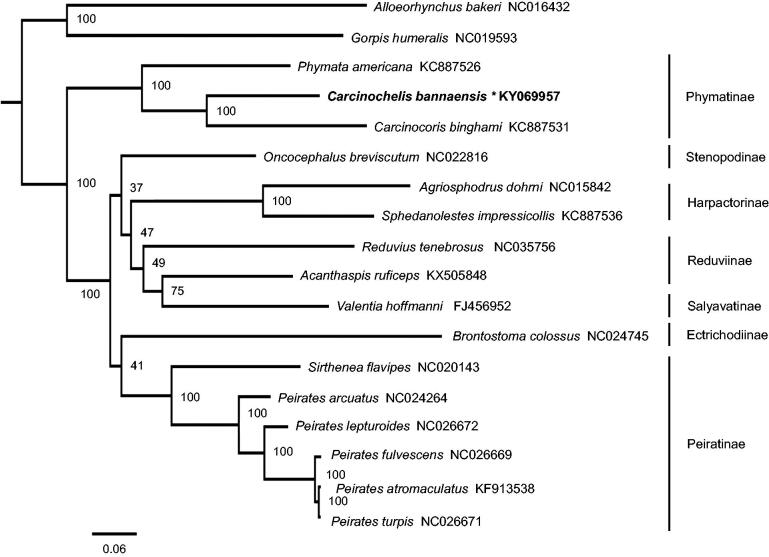
Phylogenetic relationship of *Carcinochelis bannaensis* and other 17 species among Reduviidae. Phylogenetic tree was inferred from ML analysis of the 13 protein-coding genes and two rRNAs genes (12,697bp) and generated by IQ-TREE 1.6.5 (Trifinopoulos et al. 2016). The numbers beside the nodes are percentages of 1000 bootstrap values. Alphanumeric terms indicate the GenBank accession numbers.
